# Troponin T and Survival following Cardiac Surgery in Patients Supported with Extracorporeal Membrane Oxygenation for Post-Cardiotomy Shock

**DOI:** 10.3390/diagnostics14010045

**Published:** 2023-12-25

**Authors:** Małgorzata Celińska-Spodar, Marta Załęska Kocięcka, Ilona Kowalik, Piotr Kołsut, Ewa Sitkowska-Rysiak, Jarosław Szymański, Janina Stępińska

**Affiliations:** 1 Department of Anaesthesiology and Intensive Care, The National Institute of Cardiology, 04-628 Warsaw, Poland; 2 Department of Mechanical Circulatory Support and Transplantation, Department of Heart Failure and Transplantology, The National Institute of Cardiology, 04-628 Warsaw, Poland; 3 Clinical Research Support Center, The National Institute of Cardiology, 04-628 Warsaw, Poland; 4 Department of Cardiac Surgery and Transplantation, The National Institute of Cardiology, 04-628 Warsaw, Poland; 5 Department of Cardiac Intensive Care, The National Institute of Cardiology, 04-628 Warsaw, Poland

**Keywords:** cardiogenic shock, heart failure, cardiac surgery, post-cardiotomy, VA-ECMO

## Abstract

Background: While troponin is an established biomarker of cardiac injury, its prognostic significance in post-cardiotomy cardiogenic shock patients supported by venoarterial extracorporeal membrane oxygenation (PCCS–VA-ECMO) remains unclear. Objective: This study aimed to assess the correlation between early post-operative troponin T levels and both short-term and long-term mortality outcomes in this cohort. Methods: We evaluated 1457 troponin T measurements from 102 PCCS–VA-ECMO patients treated from 2013 to 2018 at a specialized cardio-surgical and transplantation center. Emphasis was placed on troponin concentrations at 24–48 h post-surgery, post-VA-ECMO implantation, and peak troponin levels in relation to VA-ECMO weaning, as well as 90-day and one-year mortality. Results: No significant association was observed between troponin T levels post-VA-ECMO implantation and 90-day mortality (median: 1338 ng/L for overall, 1529 ng/L for survivors vs. 1294 ng/L for non-survivors; *p* = 0.146) or between peak troponin levels and 90-day mortality (median: 3583 ng/L for overall, 3337 ng/L for survivors vs. 3666 ng/L for non-survivors; *p* = 0.709). Comprehensive multivariate models showed no correlation between troponin levels and various mortality endpoints. Notably, age, procedure urgency, type, LVEF pre-surgery, Euroscore II, prior cardiac arrest, and VA-ECMO duration were not linked with troponin release. Hemodiafiltration emerged as the strongest mortality risk factor [HR 2.4]. Conclusions: Isolated early Troponin T release and peak troponin T were not associated, while organ complications were linked with VA-ECMO weaning or short- and long-term prognosis. The results underscore the multi-organ implications of PCCS in determining survival.

## 1. Introduction

Venoarterial extracorporeal membrane oxygenation (VA-ECMO) use has been increasing in popularity steadily in recent decades as a powerful mechanical circulatory support (MCS) in patients with refractory cardiogenic shock.

Amid a broad spectrum of possible specific indications for VA-ECMO implantation, post-cardiotomy cardiogenic shock following heart surgery (PCCS) is currently the most frequent one in both the United States and Europe [1,2].

As the data suggest, VA-ECMO may be associated with improved survival in this group rather than a nearly certain fatal outcome if left without MCS. VA-ECMO provides hemodynamic balance until the recuperation of cardiac function or until heart transplantation or other forms of durable MCS can be introduced. However, the use of VA-ECMO creates a risk of prolonging life-sustaining therapy in patients in whom further treatment is not indicated [3,4,5]. Therefore, intensive ongoing research focuses on identifying explicit biomarkers of cardiac recovery that could be used in the management of the therapy plan of the ECMO recipient, including early estimated triage to the possibility of weaning of ECMO, heart transplantation/LVAD candidacy or avoiding unnecessary prolongation of life-sustaining therapy [6,7].

However, despite decades of experience with PCCS–ECMO and extensive research on various aspects of VA-ECMO management, the decision-making process remains troublesome, the outcome is frequently uncertain, and no significant reduction in early mortality has been achieved [5,8,9].

One of the main reasons is the complexity of the pathophysiology of post-cardiotomy shock after heart surgery. Primary factors leading to PCCS may be of heterogenous origin and include, among others, direct surgical damage to the heart, ischemia–reperfusion injury, inflammatory response to extracorporeal circulation, or volume disturbances. The advantage of damaging factors over preservative mechanisms and efforts leads to post-operative myocardial stunning or permanent insult to the heart muscle, leading to secondary cardiogenic shock in its most severe manifestation [7,10].

Outside of the PCCS–ECMO population, biomarkers are widely used in optimizing the diagnostics and therapeutical management across the broad spectrum of cardiac and cardiosurgical patients. Troponin is the most widely available, used, and investigated cardiac biomarker of myocardial damage [11,12,13,14]. Consequently, it makes an appealing candidate because it is easily accessible, inexpensive, and offers a straightforward interpretation. Nowadays, troponin is also routinely evaluated in PCCS–ECMO patients to assess the degree of myocardial injury, despite the lack of prognostic evidence in this subset of post-cardiotomy patients. Multiple papers have tried to provide insights into the risk factors of mortality in PCCS–ECMO patients [3,4,5,15]. Unfortunately, none of the papers directly address the specific research question regarding whether troponin could be a prognostic marker in adult PCCS–ECMO.

The two published studies addressing the predictive value of troponin in the ECMO population concerned VA-ECMO following cardiogenic shock of heterogenous etiology and complicating myocardial infarction [16,17].

Our study aimed to investigate the troponin concentration 24–48 h after cardiac surgery in patients subsequently supported with VA-ECMO, troponin concentration 24–48 h following VA-ECMO implantation, peak troponin concentration in patients with PCCS–ECMO and its relationship with the likelihood of heart recovery (weaning of ECMO), as well as 90-day and one-year mortality, in patients treated in a tertiary cardiosurgical, heart transplant, and mechanical circulatory support center.

## 2. Material and Methods

### 2.1. Study Design

**Figure 1:**
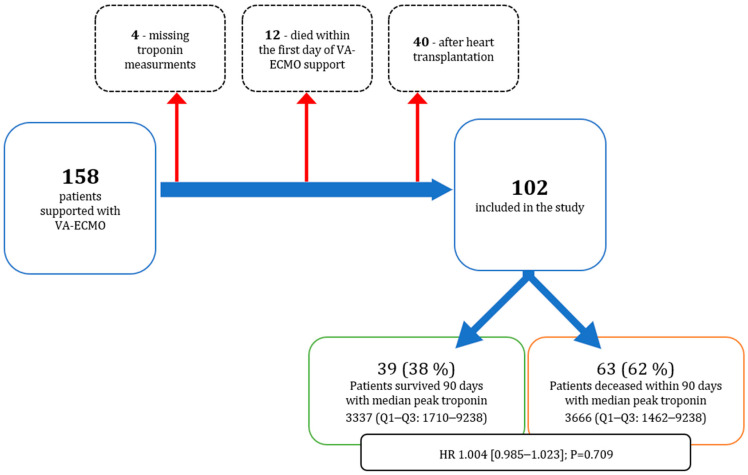
Flowchart of the study.

This study is a retrospective analysis conducted on all patients who received VA-ECMO due to cardiocirculatory failure following an open-heart procedure in our center from 2013 to 2018 (Figure 1).

According to our institutional protocol, in the post-cardiotomy setting, VA-ECMO implantation is considered by the ECMO team (including a cardiac surgeon, an anesthesiologist, and a cardiologist/intensivist) in the case of failure to wean from cardiopulmonary bypass after cardiac surgery or cardiogenic shock refractory to intensive volume and pharmacological treatment in the post-operative course. Implantation of mechanical circulatory support, including VA-ECMO, proceeds if either cardiac injury is considered reversible or a durable left ventricular assist device placement or heart transplantation is possible.

The entire troponin data were included in the analysis, from the post-surgical measurement in the first 24–48 h, through the ECMO initiation and support phase and its discontinuation or patient death. Ordinarily, the first blood test for troponin was sampled directly after leaving the operating room and repeated every 2 to 24 h, depending on clinical status and the signs of post-operative myocardial injury. For statistical purposes, we identified troponin results sampled between 24 and 48 h after surgery, 24 and 48 h after VA-ECMO implantation, and peak troponin value during VA-ECMO support. Other variables collected included demographical information, pre-implant clinical profile, type of procedure, complications of therapy, occurrence of weaning, and short- and long-term mortality. The primary outcome was all-cause mortality during VA-ECMO support, 90 days, and one year after VA-ECMO implantation. The observation period was censored to one year. Therefore, the follow-up time for survival was 12 months or 90 days in 90-day analyses, and for death, it was the time to death. Patients were excluded if they underwent a heart transplant before ECMO support, if the ECMO run was shorter than 24 h, or if troponin results were unavailable. Approval for the current study was obtained from the National Institute of Cardiology Institutional Science and Ethical Board (IK-NPIA-0021-88/1744/18).

### 2.2. Statistical Analysis

The study was designed to test the relationship between troponin values post-operatively and 24 to 48 h post-VA-ECMO implantation, peak troponin measurement during VA-ECMO therapy with the possibility of weaning from VA-ECMO, as well as 90-day and one-year mortality.

Descriptive analysis was provided to describe clinical and laboratory variables and to display distinctions between ECMO, 90-day, and one-year survivors and non-survivors as well as for comparison of groups with failure to wean from cardiopulmonary bypass (ECMO-CBP) and cardiocirculatory decompensation in the post-operative period (ECMO-ICU). Categorical variables were presented as counts and percentages and further analyzed with Pearson’s chi-square test for binary comparison or Cochran–Mantel–Haenszel Modified Ridit Scores for non-time-to-event categorical variables with >2 categories. According to their distribution, continuous variables were reported as median (Q1: 25th–Q3: 75th percentiles) and compared using the Wilcoxon rank-sum test. The relationships between the magnitude of troponin release and a series of clinical, peri-, intra-, and post-operative features were analyzed with Spearman’s correlation coefficient test for continuous variables and Wilcoxon rank-sum test for categorical variables. Univariable proportional hazards Cox regression was used to assess the impact of specific clinical and laboratory variables on mortality.

Variables identified as significant were entered into a multivariable model with backward selection. We forced the presence of troponin in the final model (all 3 variables together in one model (no co-lineality) but also each in a separate one) as it is the main subject of our analyses. Hazard ratios (HRs) with a 95% confidence interval (CI) were calculated. The goodness of fit of the models was assessed with Harrell’s C-index. A two-tailed *p*-value of <0.05 was considered statistically significant. The statistical analysis was conducted using the SAS software version 9.4 (SAS Institute Inc., Cary, NC, USA).

## 3. Results

### 3.1. Patient Characteristics and ECMO Treatment

Between January 2013 and June 2018, 11,874 patients underwent cardiac surgery at our center. Of these, VA-ECMO was initiated for 158 patients (1.3%) primarily due to complications from cardiopulmonary bypass (CPB) weaning or cardiocirculatory decompensation post-operatively. Exclusions were made for post-heart transplantation VA-ECMO patients (*n* = 40), those deceased within the first 24 h of VA-ECMO support (*n* = 12), and cases with missing troponin values (*n* = 4). Our final cohort consisted of 102 patients, representing 1457 troponin measurements (Figure 1).

The cohort had a median age of 64.1 years with 57.8% males. Medical histories indicated 52.9% with chronic kidney disease, 30.4% with diabetes, and 5.9% with prior neurological events. Notably, 16.7% experienced cardiac arrest within the 24 h preceding VA-ECMO. Surgical urgency was distributed fairly evenly: emergent (36.3%), urgent (31.4%), and elective (32.3%). The majority underwent either a single non-CABG procedure (41.2%) or dual procedures (32.3%). A less frequent indication for surgery was three procedures combined (13.7%) and isolated CABG (12.7%).

Half of the patients (52%, *n* = 53) were transitioned to VA-ECMO post-operatively for post-cardiotomy low cardiac output syndrome or post-operative cardiac arrest after a median ICU stay of 46 h. The remaining 48% (*n* = 49) were shifted intraoperatively to central VA-ECMO due to CPB weaning failures. VA-ECMO support lasted a median of 227 h. Slightly over two-fifths of patients (41.2%, *n* = 42) had concomitant intra-aortic balloon pump (IABP) support as a left ventricle unloading strategy.

ECMO-related complications are predominantly involved with nearly three-quarters of patients (73.5%) experiencing at least one clinically overt bleeding episode. Acute kidney injury led to continuous renal replacement therapy (CRRT) in 56.9% of patients, 54.9% acquired infections, 31.4% required prolonged mechanical ventilation (greater than 10 days), and 10.8% suffered acute brain injuries (including TIA, ischemic stroke, or ICH) while on VA-ECMO.

A total of 39% of patients died during VA-ECMO after a median of 5.6 days, and the all-cause in-hospital mortality was 62.7%. For patients who survived to hospital discharge (37.3%), survival after one year was 86.8%.

Univariable Cox regression identified the need for CRRT during VA-ECMO to be significantly associated with mortality on ECMO, on 90-day and one-year follow-up after VA-ECMO implantation. A higher score on the pre-operative Euroscore 2 assessment was tied to one-year mortality (*p* = 0.048) but not short-term mortality during ECMO support or 90-day mortality (*p* = 0.655 and *p* = 0.096, respectively).

Notably, the adjuvant use of IABP support along with VA-ECMO was tied to a significantly higher rate of survival during the VA-ECMO support phase and the 90 days following ECMO initiation (*p* = 0.03 and *p* = 0.035, respectively).

Patient characteristics at baseline, interventions, and complications of VA-ECMO for the entire cohort and stratified by mortality during VA-ECMO, as well as 90 days and one year thereafter, are presented in Table 1.

### 3.2. Primary Outcome—Troponin Concentration and Mortality

**Figure 2:**
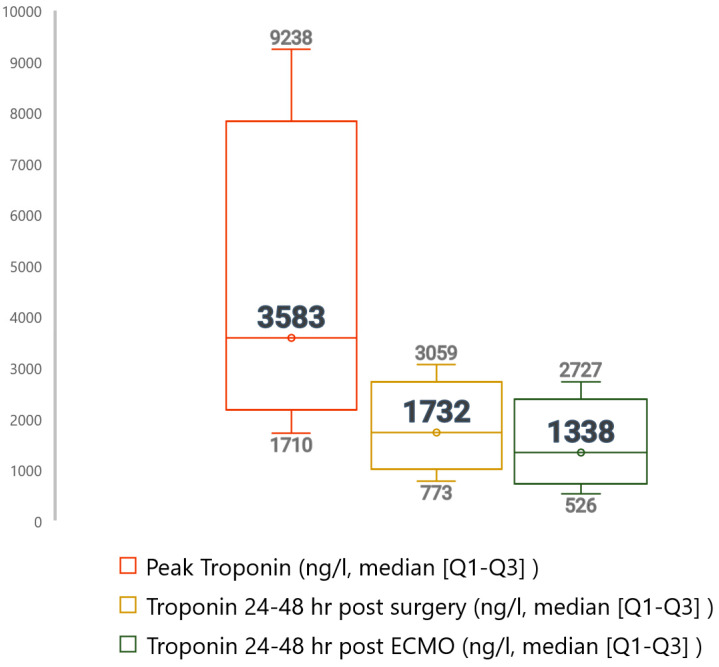
Troponin 24–48 h following VA-ECMO implantation, 24–48 h following surgery, and peak values presented as medians and quartiles (IQR 1–3) of troponin in subgroups.

There was no statistically significant association between troponin values and mortality at any analyzed endpoints (VA-ECMO support, 90 days, and one-year post-VA-ECMO) (Supplementary Figures S1–S3). The median troponin concentration 24–48 h post-VA-ECMO was 1338 ng/L, differing insignificantly between 90-day survivors and non-survivors (1295 vs. 1112 ng/L, *p* = 0.256). The median peak troponin value during VA-ECMO support was 3583 ng/L, with no discernible variance between survival outcomes (Figure 2).

Similarly, no significant association was found between troponin measured 24–48 h post-surgery in patients undergoing implantation in the post-operative period (Table 1).

Table 2 depicts the comparison of groups with failure to wean from cardiopulmonary bypass (ECMO–CPB) (48%) and with cardiocirculatory decompensation in the post-operative period (ECMO–ICU) (52%). The median time interval from the end of surgery to VA-ECMO implantation in the latter group was 1.9 days.

Patients implanted in the post-operative period had higher values of troponin 24–48 h following initial surgery than those who transitioned directly from CPB, with no statistical significance (1978 vs. 1338 ng/L, *p* = 0.083). Conversely, peak troponin was higher for the latter (4753 vs. 2785 ng/L, respectively, *p* = 0.115).

In the subsequent step of the analysis, we conducted backward multivariable Cox regression analyses which included troponin values at previously defined time points (24–48 h post-surgery, 24–48 h post-VA-ECMO implantation, and peak value) combined with the variables that showed significance in the univariable Cox regression analysis: duration of VA-ECMO support, use of CRRT, use of IABP, Euroscore 2 result, and the clinically important parameters: time of mechanical ventilation, bleeding episode, elective mode of surgery, central cannulation, ABI, pre-operative LVEF, failure to wean from CPB, time interval to ECMO implantation, BMI and age.

The initial dependent variables: IABP, Euroscore 2 bleeding episode, elective mode of surgery, central cannulation, ABI, pre-operative LVEF, failure to wean from CPB, time interval to ECMO implantation, BMI and age, dropped out during backward elimination because they did not reach statistical significance (*p* > 0.05).

To test the primary question of the study, troponins were kept until the final model, despite not reaching statistical significance.

The final three multivariable models: for mortality during VA-ECMO explanation, 90-day and one-year mortality, confirmed a lack of association between troponin concentration and mortality at all specific end-time points (Table 3).

Subsequently, we performed the investigation of the relationships between the magnitude of troponin release and a series of clinical, peri-, intra- and post-operative features, which results are presented in Table 4. Patients who underwent elective surgery had significantly higher values of troponin 24–48 h after surgery vs. those operated in non-elective mode (combined urgent and emergent). Additionally, the analysis revealed significantly higher values of peak troponin in patients operated in non-urgent vs. urgent mode (5760 ng/L vs. 2553 ng/L, respectively, *p* = 0.022).

We did not find any other significant association between the remaining clinical variables included in the study, such as age, type and number of procedures, pre-operative LVEF, the Euroscore II results, cardiac arrest prior to VA-ECMO implantation, duration of VA-ECMO support or use of IABP, and the extent of troponin release (Table 4).

## 4. Discussion

According to our study, patients suffering from post-cardiotomy low cardiac output syndrome are characterized by a very high cardiac troponin release similar to the values observed during the gold-standard indication for troponin measurement–acute myocardial infarction or heart failure decompensation. However, we did not find any predictive relation between the cardiac troponin values 24 h after ECMO implantation or the maximum troponin plasma concentration and the possibility of cardiac regeneration or short- or long-term mortality in patients with VA-ECMO support in the post-cardiotomy phase.

PCCS complicates approximately 2–5% of cardiac surgeries and is followed by elevated mortality rates [2,18].

Across the recent decades, VA-ECMO has been exponentially implemented as a life-saving procedure in PCCS, improving survival rates in refractory to standard therapy cardiogenic shock. However, despite growing evidence and experience with ECLS worldwide, mortality remains high, with survival to hospital discharge in only approximately 42% of cases (2997/7185), according to a recent ELSO registry analysis of the post-cardiotomy cohort [5].

VA-ECMO implemented in PCCS offers time for bridging to the recovery of the potentially reversible heart injury following cardiac procedure or, if recovery does not occur, to the decision to advance to more durable support modes.

However, in the course of critical illness of patients with PCCS, the decision-making process is rapid and troublesome, and the outcome is often unforeseeable. Defining a single prognostic marker of myocardial recovery amid multiple concurrent factors appears convenient in empowering decisions regarding the weaning of ECMO or durable LVAD candidacy, or conversely, in limiting prolonged ECMO runs in patients in whom further treatment is not indicated.

Nevertheless, the reliability of prognostic biomarkers, tested across patients outside of the ECLS setting, is vastly unknown in patients on ECLS. Cardiac troponins T and I are well-established, fundamental elements of the diagnostic cascade in diagnosing myocardial infarction, determining the extent of myocardial injury, and establishing the short- and long-term survival prognosis in acute and chronic heart failure [10,11,13,19].

The interpretation of cardiac troponin levels following cardiac surgical procedures is less straightforward, as various confounding factors boosting troponin release are involved, such as underlying pathology and comorbidities, the complexity and duration of the procedure, as well as perioperative complications. Yet, the value of troponin after cardiac surgery has been demonstrated to have a prognostic impact on long-term mortality and major cardiac events across a series of studies [14,20].

Based on the study population of 1356 patients, Croal et al. [14] demonstrated that cTnI levels 24 h after cardiac surgery are strong and independent predictors of short- and long-term mortality regardless of other perioperative factors.

It has not yet been explored if the significance of cardiac troponin levels, even if adapted from the general post-cardiotomy population, can be fitted to estimate the prognosis of patients supported with ECMO, as few critical distinctions can be marked in the ECMO subgroup that can diminish the validity of this universal biomarker.

According to various single-center reports, patients requiring ECLS following cardiac surgery are a slim fraction of all post-cardiotomy patients, between 0.4% and 3.7% [7]. However, they are at the end of the spectrum of severity of post-cardiotomy complications. Compared with the general post-cardiotomy population, PCCS–ECMO recipients are characterized as being in a relatively more serious condition, with a higher incidence of pre-operative renal insufficiency and coronary artery disease, prior myocardial infarction, greater extent of left ventricular (LV) dysfunction, preceding cardiac surgery, and urgent or emergent operative status [7]. The mortality of patients with PCCS–ECMO is higher than in other VA-ECMO indications for cardiac failure [7,9,10].

ECMO implementation is a powerful modifier of hemodynamic conditions, providing sufficient cardiac output, limiting end-organ damage, and reducing exposure to high-dose inotropes [7,10,21]. However, complications adherent to the ECMO physiology and mode of action are common and frequently detrimental to survival. Life-threatening incidents such as bleeding, severe renal injury, and neurological damage, are more frequent in PCCS–ECMO compared to other ECMO indications, which may be caused by the preceding cardiac surgery and cardiopulmonary bypass [7,21]. In a recent ELSO registry analysis of 7185 patients treated with VA-ECMO for post-cardiotomy shock, Kowalewski et al. reported kidney failure in 48.9%, surgical site bleeding in 26.4%, and neurologic complications in 9.1% of patients. The complications were significantly associated with hospital mortality in multivariable analysis [5].

As it has been established that the quantity of cardiac troponin reflects the degree of myocardial injury and, less specifically, critical state, sepsis, and end-organ injury [13,22], it is not surprising that all patients undergoing ECMO support for PCCS will have elevated cardiac troponin levels post-operatively.

The open question remains if its predictive power surpasses the pivotal impact of adverse clinical events on survival.

Only a few studies have examined the value of troponin as a biomarker in predicting cardiac recovery in patients with cardiogenic shock supported with VA-ECMO, providing discordant results. Nevertheless, none of them focused specifically on the adult PCCS–ECMO cohort.

In a study conducted by Ruffer and colleagues, troponin I levels were assessed in 34 pediatric patients who were placed on VA-ECMO following surgery for congenital heart defects. Interestingly, the peak levels of troponin I observed 24 h after the operation did not significantly differ between patients who survived and those who did not. However, a stable troponin I level at the 48 h mark, without any subsequent decline, was identified as a potential marker of poor prognosis, possibly suggesting irreversible myocardial damage [23]. In the adult VA-ECMO population, Lyut et al. assessed troponin release and its kinetics on treatment days 1, 3, or 7 in 41 consecutive patients with cardiogenic shock of heterogenous etiology treated with VA-ECMO, but found no predictive value in terms of cardiac recovery [16]. In contrast, based on the review of 62 consecutive patients who received VA-ECMO for refractory cardiogenic shock complicating acute myocardial infarction, North et al. showed that peak troponin of 400 ng/mL, with a specificity of 71% and a sensitivity of 98%, correctly classified the ability of 90% of patients to be weaned or not weaned off ECMO [17].

**Figure 3:**
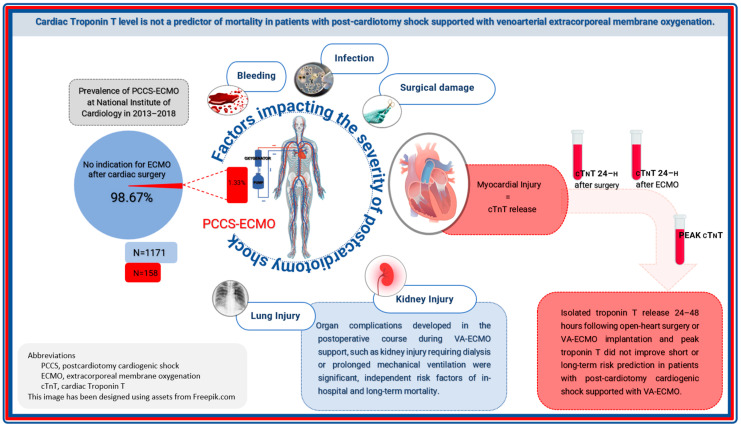
Isolated troponin T release 24–48 h following open-heart surgery or VA-ECMO implantation and peak troponin T do not improve short- or long-term risk prediction in patients with PCCS supported with VA-ECMO. On the other hand, organ complications developed post-operatively during VA-ECMO support, such as kidney injury requiring dialysis or prolonged mechanical ventilation, were significant independent risk factors of in-hospital and long-term mortality.

Still, given that patients after open-heart surgery have very different risk characteristics among themselves, and even more than patients without preceding operation, including different baseline parameters and additional inherent risk associated with intra- and post-operative course, it is justifiable to statistically treat PCCS–ECMO and non-post-cardiotomy ECMO groups separately to reduce data heterogeneity. Cardiovascular insult during or following heart surgery, causing drastic impairment of cardiac performance and cardiogenic shock, is commonly associated with multi-organ damage and general critical illness which may explain the lack of statistical association with mortality attributable to early perioperative troponin (Figure 3) [15]. 

## 5. Conclusions

Isolated troponin T release 24–48 h following open-heart surgery or VA-ECMO implantation and peak troponin T do not improve short- or long-term risk prediction in patients with PCCS supported with VA-ECMO. On the other hand, organ complications developed post-operatively during VA-ECMO support, such as kidney injury requiring dialysis or prolonged mechanical ventilation, were significant independent risk factors of in-hospital and long-term mortality (Figure 3).

## 6. Strengths and Limitations

According to our extensive data search, this study is the first to analyze troponin concentrations in post-cardiotomy patients supported with VA-ECMO.

The most outstanding drawback of the study is the limitation of the study sample to one center and its retrospective design. The sample size of our study is confined, which can affect the statistical power and the robustness of our findings. The outcomes and conclusions of this study should be viewed in this context and treated with caution. Our findings, derived from a specialized center, may not be universally applicable due to variations in patient demographics and treatment protocols across different healthcare settings. While our findings contribute to the existing body of knowledge, further prospective studies with larger participant pools are recommended to validate and potentially expand upon our results.

## Figures and Tables

**Table 1:** Clinical and demographical parameters and their association with mortality: during VA-ECMO support, within 90 days, and one year after VA-ECMO initiation.

Mortality (No./Total No. (%))			ECMO 40/102 (39.2)		90-Day 63/102 (61.8)		One-Year 68/102 (66.7)	
Factor	Results	Unit	HR (95% CI)	*p*	HR (95% CI)	*p*	HR (95% CI)	*p*
Troponin 24–48 h post-surgery	1732 (773–3059)	ng/L median (Q1–Q3)	0.991 (0.939–1.047)	0.753	0.960 (0.908–1.014)	0.146	0.980 (0.936–1.025)	0.375
Troponin 24–48 h post-ECMO	1338 (526–2727)	ng/L median (Q1–Q3)	0.997 (0.948–1.049)	0.919	0.969 (0.918–1.023)	0.256	0.983 (0.940–1.029)	0.463
Peak troponin	3583 (1710–9238)	ng/L median (Q1–Q3)	1.010 (0.991–1.030)	0.298	1.004 (0.985–1.023)	0.709	1.008 (0.991–1.025)	0.367
Duration of ECMO	227 (121–377)	hours median (Q1–Q3)	0.999 (0.997–1.000)	0.146	0.999 (0.998–1.000)	0.093	1.000 (0.999–1.000)	0.333
Mechanical ventilation time	76.2 (33.5–229.4)	hours median (Q1–Q3)	1.00 (0.999–1.001)	0.672	1.00 (0.999–1.001)	0.784	1.000 (1.000–1.001)	0.311
Age	64.1 (56.7–69.5)	years median (Q1–Q3)	1.015 (0.991–1.039)	0.223	1.016 (0.996–1.035)	0.112	1.015 (0.996–1.033)	0.120
LV EF prior ECMO	25 (15–40)	% median (Q1–Q3)	1.004 (0.987–1.021)	0.669	0.999 (0.986–1.013)	0.893	0.997 (0.984–1.011)	0.671
EUROSCORE II	8.3 (3.8–18.1)	% median (Q1–Q3)	1.776 (0.143–22.124)	0.655	5.006 (0.752–33.32)	0.096	6.223 (1.012–38.26)	**0.048**
Mode of surgery elective vs. non-elective (emergent + urgent)	33 (32.3)	*n*, %	1.702 (0.909–3.187)	0.096	1.165 (0.690–1.967)	0.568	1.032 (0.617–1.727)	0.904
Procedures (1 vs. more than 1)	55 (53.9)	*n*, %	0.925 (0.497–1.721)	0.806	1.002 (0.610–1.644)	0.995	1.085 (0.672–1.752)	0.738
Central cannulation	70 (68.6)	*n*, %	0.728 (0.380–1.395)	0.339	0.838 (0.493–1.426)	0.515	0.822 (0.494–1.367)	0.450
ECMO-CPB	49 (48.0)	*n*, %	0.586 (0.309–1.113)	0.102	0.890 (0.543–1.460)	0.645	0.822 (0.510–1.325)	0.421
IABP	42 (41.2)	*n*, %	0.463 (0.231–0.928)	**0.030**	0.568 (0.336–0.960)	**0.035**	0.610 (0.370–1.005)	0.052
CRRT	58 (56.9)	*n*, %	2.820 (1.374–5.787)	**0.005**	2.603 (1.501–4.513)	**<0.001**	2.664 (1.572–4.515)	**<0.001**
Any Bleeding	75 (73.5)	*n*, %	1.084 (0.530–2.219)	0.825	1.435 (0.779–2.644)	0.246	1.500 (0.832–2.704)	0.178
ABI	11 (10.8)	*n*, %	1.606 (0.709–3.636)	0.256	1.385 (0.682–2.811)	0.368	1.322 (0.654–2.674)	0.437

The second column shows the results as medians and quartiles or as counts (percentages). The *p*-values for Wald chi-square statistics were derived from univariable Cox ph regression; HRs (95% CI) are calculated for troponin expressed in micrograms (μg/L). Abbreviations: Troponin—High-sensitivity cardiac troponin T level—ng/L; ECMO, venoarterial extracorporeal membrane oxygenation; CPB, cardiopulmonary bypass; ECMO-CPB, failure to wean from CPB; LV EF, left ventricular ejection fraction; CRRT, continuous renal replacement therapy; ABI, acute brain injury; IABP, intra-aortic balloon pump. Grey background color indicate statistical significance (*p* < 0.05).

**Table 2:** Comparison of groups with failure to wean from cardiopulmonary bypass (ECMO-CPB) (48%) and cardiocirculatory decompensation in the post-operative period (ECMO-ICU) (52%).

Variables (No./Total No. (%))	ECMO-CBP 49/102 (48.0)	ECMO-ICU 53/102 (52.0)	*p*
Pre-operative and demographic data			
Age, years, median (Q1–Q3)	64.1 (56.7–66.9)	64.0 (57.4–70.0)	0.693
Body mass index, kg/m^2^, mean ± SD	27.1 ± 4.5	26.1 ± 4.0	0.246
Male, *n*, %	30 (61.2)	29 (54.7)	0.506
CKD, *n*, %	26 (53.1%)	28 (52.8%)	0.981
DM, *n*, %	12 (24.5%)	19 (35.9%)	0.213
ABI, *n*, %	1 (2.0%)	5 (9.4%)	0.207
LV EF prior VA ECMO, %, median (Q1–Q3)	30.0 (15–45)	20.0 (15–40)	0.293
Euroscore II, %, median (IQR)	10.7 (4.4–23.1)	6.7 (3.0–12.0)	0.051
Peri-procedural data			
Timing of the intervention			
Elective	15 (30.6%)	18 (34.0%)	**0.004**
Urgent	9 (18.4%)	23 (43.4%)	
Emergency	25 (51.0%)	12 (22.6%)	
CABG vs. no-CABG, *n*, %			
Single vessel CABG	3 (6.1%)	10 (18.9%)	0.097
Multi vessels CABG	24 (49.0%)	18 (34.0%)	
Another cardiac surgery	22 (44.9%)	25 (47.2%)	
Number of procedures			
1	27 (55.1%)	28 (52.8%)	0.935
2	15 (30.6%)	18 (34.0%)	
3	7 (14.3%)	7 (13.2%)	
Cardiac arrest prior VA-ECMO, *n*, %	3 (6.1%)	14 (26.4%)	0.006
Post-procedural data			
LV EF post-VA-ECMO, %, median (Q1–Q3)	15.0 (10–20)	15.0 (10–30)	0.697
IABP, *n*, %	22 (44.9%)	20 (37.7%)	0.463
Any bleeding, *n*, %	40 (81.6%)	35 (66.0%)	0.074
ABI event during ECMO, *n*, %	4 (8.2%)	7 (13.2%)	0.412
CRRT during ECMO, *n*, %	22 (44.9%)	36 (67.9%)	0.019
Extubation on ECMO, *n*, %	29 (59.2%)	26 (49.1%)	0.305
Central cannulation, *n*, %	38 (77.5%)	32 (60.4%)	0.062
MV time, hours, median (Q1–Q3)	68.0 (33.0–220.0)	92.6 (34.3–241.1)	0.474
ICU stay, days, median (Q1–Q3)	21.9 (12.0–36.3)	16.6 (8.3–34.0)	0.484
Tracheostomy, *n*, %	5 (10.6%)	7 (13.2%)	0.693
Infection, *n*, %	25 (51.0%)	31 (58.5%)	0.449
Duration of VA-ECMO, hours, median (Q1–Q3)	238 (141–312)	211 (98–362)	0.547
Troponin concentrations			
Troponin 24–48 h after surgery (ng/L, median (Q1–Q3))	1338 (552–2727)	1978 (1061– 3337)	0.083
Time from surgery to ECMO, days, median (Q1–Q3)	0.00	1.9 (0.5–3.3)	
Peak Troponin, ng/L, median (Q1–Q3)	4753 (2058–13,900)	2785 (1462–6904)	0.115
Outcomes			
Mortality			
During VA-ECMO, *n*, %	15 (30.6)	25 (47.2)	0.087
90-day mortality, *n*, %	30 (61.2)	33 (62.3)	0.914
One-year mortality, *n*, %	31 (63.3)	37 (69.8)	0.483

Results are presented as counts (percentages) or median (Q1–Q3). The *p*-values were derived from the Pearson’s chi-square test for binary comparison, appropriate CMH—statistics for nominal or ordinary data, and Wilcoxon rank-sum test for continuous variables. Abbreviations: Troponin—High-sensitivity cardiac troponin T level—ng/L; ECMO, venoarterial extracorporeal membrane oxygenation; CBP, cardiopulmonary bypass; ICU, Intensive care unit; CABG, coronary artery bypass grafting; CKD, chronic kidney disease; DM, diabetes mellitus; ABI, acute brain injury; CRRT, continuous renal replacement therapy; LV EF, left ventricular ejection fraction; IABP, intra-aortic balloon pump; MV time, total mechanical ventilation time. Grey background color indicate statistical significance (*p* < 0.05).

**Table 3:** Factors associated with mortality: during VA-ECMO support, 90 days, and one year after VA-ECMO initiation in multivariable analysis.

Mortality (No./Total No. (%))	ECMO 40/102 (39.2)		90-Day 63/102 (61.8)		One-Year 68/102 (66.7)	
Variables	HR (95% CI)	*p*	HR (95% CI)	*p*	HR (95% CI)	*p*
Troponin 24–48 h post-surgery	0.924 (0.746–1.144)	0.468	0.886 (0.743–1.057)	0.179	0.960 (0.845–1.091)	0.535
Troponin 24–48 h post-ECMO	1.077 (0.876–1.325)	0.480	1.062 (0.902–1.252)	0.469	1.001 (0.881–1.138)	0.984
Peak Troponin	1.006 (0.980–1.033)	0.665	1.012 (0.986–1.038)	0.369	1.013 (0.989–1.037)	0.296
CRRT	2.456 (1.142–5.284)	**0.021**	2.716 (1.497–4.928)	**0.001**	2.491 (1.410–4.410)	**0.002**
ECMO hours	0.993 (0.989–0.997)	**<0.001**	0.996 (0.993–0.998)	**0.013**	0.996 (0.994–0.999)	**0.002**
MV time	1.006 (1.002–1.011)	**0.002**	1.003 (1.001–1.006)	**0.012**	1.003 (1.001–1.006)	**0.006**
Harrell’s Concordance (95% CI)	0.799 (0.740–0.858)		0.754 (0.696–0.812)		0.731 (0.678–0.784)	

The *p*-values for Wald chi-square statistics were derived from a multivariable Cox ph regression model with backward selection and forced the presence of troponin. HRs (95% CI) are calculated for troponin expressed in micrograms (μg/L). Abbreviations: ECMO, venoarterial extracorporeal membrane oxygenation; Troponin—High-sensitivity cardiac troponin T level; CRRT, continuous renal replacement therapy; MV time, total mechanical ventilation time. Grey background color indicate statistical significance (*p* < 0.05).

**Table 4:** Relationships between troponin concentration and other investigated features.

Variables	Troponin 24–48 h after Surgery	*p*	Troponin 24–48 h after VA-ECMO Implantation	*p*	Peak Troponin during VA-ECMO Support	*p*
Age	−0.13	0.178	−0.12	0.226	−0.15	0.144
Body mass index	0.01	0.895	−0.07	0.494	0.110	0.271
Gender						
Male vs. Female	1754 (828–2727)	0.799	1353 (755–2449)	0.502	2824 (1710–7351)	0.116
	1702 (467–1831)		1321 (282–3891)		5817 (1380–17,560)	
Mode of surgery						
Elective (*n* = 33) vs. Non-elective (*n* = 69)	2270 (1321–3891)	**0.028**	1800 (1123–3044)	0.064	5817 (1837–12,305)	0.387
	1353 (552–2824)		1178 (319–2657)		3335 (1710–8481)	
Urgent (*n* = 32) vs. Non-urgent (*n* = 70)	1366 (712–2337)	0.206	969 (422–2311)	0.273	2553 (1416–4660)	**0.022**
	1969 (777–3808)		1376 (552–3641)		5760 (1900–15,800)	
Emergent (*n* = 37) vs. Non-emergent (*n* = 65)	1338 (336–3641)	0.358	1294 (316–3641)	0.458	5730 (1932–16,870)	0.172
	1800 (845–2948)		1353 (755–2474)		3163 (1462–8452)	
Type of surgery						
Isolated CABG (*n* = 13) vs. Rest combined (*n* = 89)	2420 (1353–3400)	0.413	1898 (257–2449)	0.960	4567 (2775–20,650)	0.246
	1600 (773–3044)		1321 (552–2727)		3337 (1502–9000)	
Single no-CABG (*n* = 42) vs. Rest combined (*n* = 60)	1359 (777–3337)	0.593	1308 (476–2075)	0.617	3641 (1321–7351)	0.343
	1880 (712–2964)		1531 (539–2776)		3532 (18,446–15,115)	
Single CABG (*n* = 13) vs. Single non-CABG (*n* = 42)	2420 (1353–3400)	0.389	1898 (257–2449)	0.820	4567 (2775–20,650)	0.231
	1359 (777–3337)		1531 (539–2776)		3532 (18,446–15,115)	
Number of procedures						
Single-procedure (*n* = 55) vs. Rest combined (*n* = 47)	1702 (777–3400)	0.987	1338 (327–2449)	0.648	3783 (1710–8452)	0.875
	1837 (668–2837)		1345 (552–2824)		2824 (1615–14,430)	
2 procedures (*n* = 33) vs. 3 procedures (*n* = 14)	1863 (797–3641)	0.346	1353 (755–4153)	0.269	2824 (1837–16,410)	0.617
	1549 (607–2747)		1234 (336 –2380)		2882 (1502–9000)	
Cardiac arrest pre-VA-ECMO						
Yes (*n* = 17) vs. No (*n* = 85)	1863 (755–3059)	0.696	1863 (755–4153)	0.391	3666 (1863–6904)	0.914
	1702 (777–3044)		1301 (526–2449)		3500 (1615–12,305)	
CRRT during ECMO						
Yes (*n* = 58) vs. No (*n* = 44)	1993 (607–3641)	0.395	1341 (316–3641)	0.898	5148 (1615–14,430)	0.272
	1490 (832–2480)		1329 (813–2237)		3073 (1773–6630)	
Euroscore II	−0.16	0.116	−0.16	0.101	−0.08	0.446
LV EF pre-VA-ECMO	−0.16	0.111	−0.15	0.126	0.13	0.186
LV EF post-VA-ECMO	−0.08	0.406	−0.11	0.255	−0.07	0.457
Delta LVEF	−0.11	0.250	−0.11	0.286	0.11	0.265
IABP during ECMO						
Yes (*n* = 42) vs. No (*n* = 60)	1795 (467–3056)	0.807	1341 (316–3044)	0.886	3532 (1856–7243)	0.663
	1655 (787–3142)		1329 (682–2692)		3641 (1457–15,115)	
Bleeding during ECMO						
Yes (*n* = 75) vs. No (*n* = 27)	1600 (730–3059)	0.320	1295 (476–3044)	0.590	3808 (1710–14,430)	0.306
	2075 (842–3337)		1529 (565–2657)		2824 (1502–6358)	
ABI during ECMO						
Yes (*n* = 11) vs. No (*n* = 91)	2420 (1837–9359)	0.151	2420 (1211–9359) 1301 (476––2474)	0.113	6904 (1837–31,170)	0.483
	1462 (755–3044)				3500 (1502–9238)	
Central cannulation						
Yes (*n* = 70) vs. No (*n* = 32)	1732 (797–3400)	0.786	1651 (731–3075)	0.226	3736 (1863–13,900)	0.235
	1590 (543–2948)		1228 (402–2227)		3080 (1255–7680)	
MV time	−0.08	0.418	−0.03	0.733	0.06	0.557
MV > 10 d						
Yes (*n* = 32) vs. No (*n* = 70)	1818 (858–2940)	0.942	1537 (802–3060)	0.552	6728 (1850–16,105)	0.105
	1651 (668–6400)		1338 (336–2474)		3135 (1462–7243)	
Duration of ICU stay	−0.00	0.991	−0.05	0.609	0.06	0.545
Tracheostomy						
Yes (*n* = 12) vs. No (*n* = 88)	1826 (200–3051)	0.535	1117 (144–2471)	0.337	10,111 (3220–17,600)	0.108
	1755 (812–3368)		1349 (682–2775)		3336 (1606–8468)	
Infection						
Yes (*n* = 56) vs. No (*n* = 46)	1795 (641–3350)	0.621	1345 (326–3358)	0.798	5276 (1704–16,185)	0.355
	1527 (797–2948)		1319 (773–2474)		3135 (1710–6742)	
Duration of VA-ECMO	0.10	0.336	0.14	0.149	0.15	0.140

Results are presented as counts (percentages) or median (Q1–Q3) and the *p*-values were derived from the Wilcoxon rank-sum U test of medians and quartiles of troponin in subgroups for qualitative variables. Spearman’s correlation coefficient test was applied to calculate quantitative variables and the *p*-value from the non-parametric test of significance of the correlation coefficient. Medians of troponins are expressed in nanograms per liter (ng/L). Abbreviations: Troponin—High-sensitivity cardiac troponin T level—ng/L; ECMO, venoarterial extracorporeal membrane oxygenation; CABG, coronary artery bypass grafting; CRRT, continuous renal replacement therapy; LV EF, left ventricular ejection fraction; Delta LV EF, difference between LV EF before surgery and after ECMO implantation; IABP, intra-aortic balloon pump, ABI, acute brain injury; MV time, total mechanical ventilation time; ICU, intensive care unit. Grey background color indicate statistical significance (*p* < 0.05).

## Data Availability

Available from the first author upon request.

## Supplementary Materials

The following supporting information can be downloaded at: https://www.mdpi.com/article/10.3390/diagnostics14010045/s1, Figure S1. Receiver Operating Characteristic (ROC) curves for troponin levels post-ECMO implantation in relation to mortality on ECMO, at 90-days and 1-year; Figure S2. Receiver Operating Characteristic (ROC) Curves for troponin levels post-surgery in relation to mortality on ECMO, at 90-days and 1-year; Figure S3. Receiver Operating Characteristic (ROC) Curves for peak troponin during ECMO support in relation to mortality on ECMO, at 90-days and 1-year.

## Abbreviations

VA-ECMO	venoarterial extracorporeal membrane oxygenation
MCS	mechanical circulatory support
PCCS	post-cardiotomy cardiogenic shock
CABG	coronary artery bypass graft
ICH	intracranial hemorrhage
TIA	transient ischemic attack
ABI	acute brain injury
CRRT	continuous renal replacement therapy
IABP	intra-aortic balloon pump
GCS	Glasgow Coma Score
