# Supplementary material for: Troponin T and Survival following Cardiac Surgery in Patients Supported with Extracorporeal Membrane Oxygenation for Post-Cardiotomy Shock
Source: Diagnostics (Basel). 2023 Dec 25;14(1):45. doi: 10.3390/diagnostics14010045 (PMC10802831; doi:10.3390/diagnostics14010045)

Supplementary Figure S1. Receiver Operating Characteristic (ROC) curves for troponin levels post-ECMO implantation in relation to mortality on ECMO, at 90-days and 1-year.

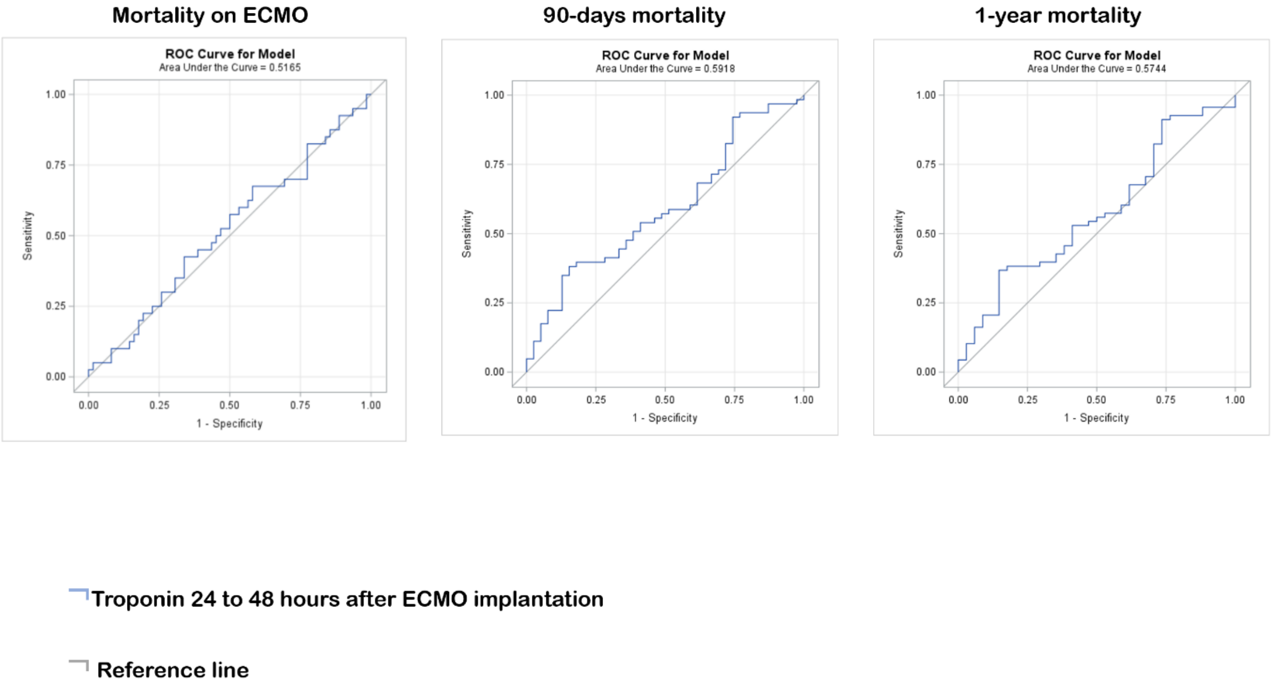

Supplementary Figure S2. Receiver Operating Characteristic (ROC) Curves for troponin levels post-surgery in relation to mortality on ECMO, at 90-days and 1-year.

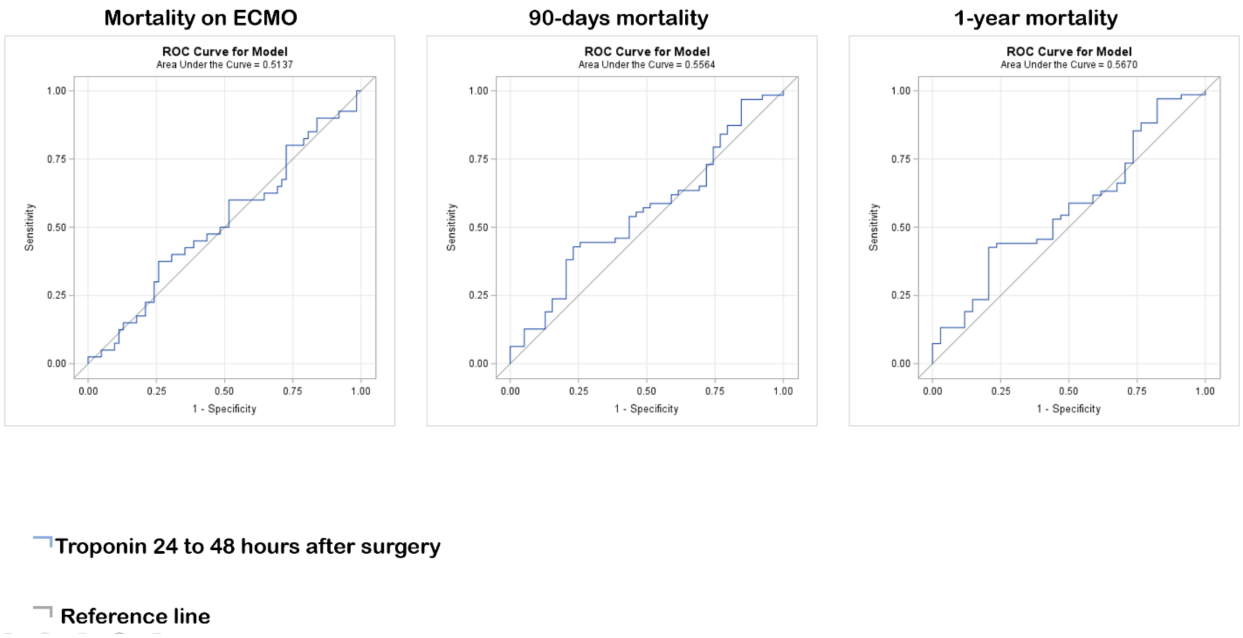

Supplementary Figure S3. Receiver Operating Characteristic (ROC) Curves for peak troponin during ECMO support in relation to mortality on ECMO, at 90-days and 1-year.

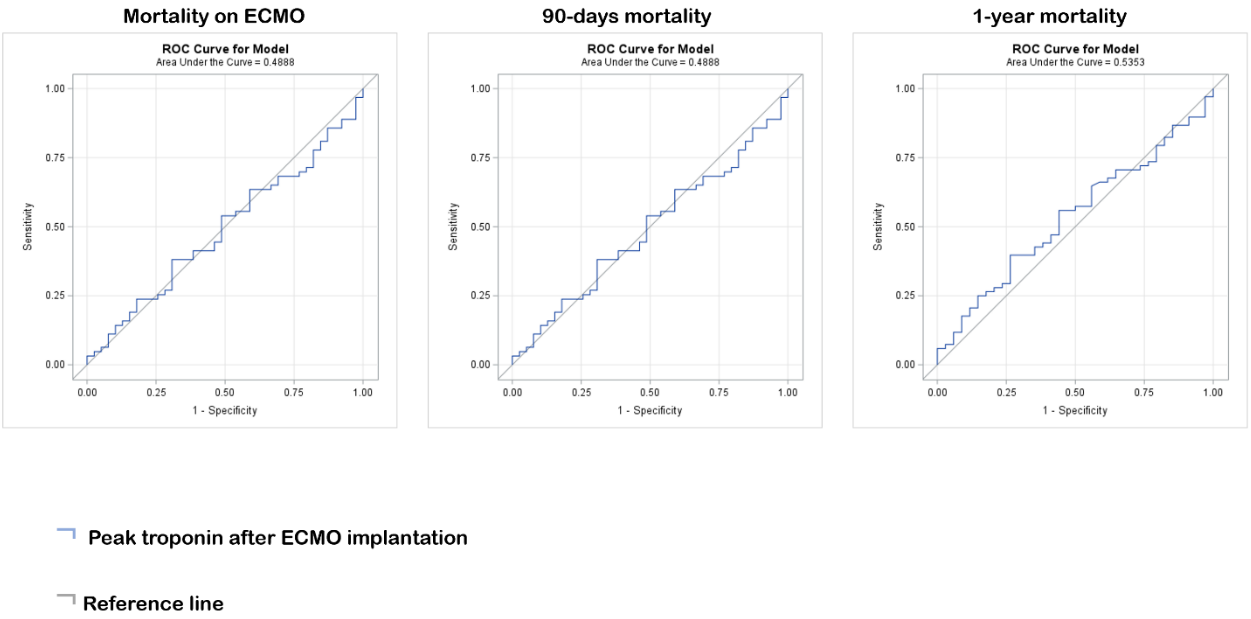

Supplement: Supplementary file 1 [file diagnostics-14-00045-s001.zip › diagnostics-2745963-supplementary.pdf]
